# Poly(l-lactide)-Based Anti-Inflammatory Responsive Surfaces for Surgical Implants

**DOI:** 10.3390/polym13010034

**Published:** 2020-12-24

**Authors:** Julia Sánchez-Bodón, Leire Ruiz-Rubio, Estíbaliz Hernáez-Laviña, José Luis Vilas-Vilela, Mª Isabel Moreno-Benítez

**Affiliations:** 1 Macromolecular Chemistry Group (LABQUIMAC), Department of Physical Chemistry, Faculty of Science and Technology, University of the Basque Country, UPV/EHU, Barrio Sarriena s/n, 48940 Leioa, Spain; leire.ruiz@ehu.eus (L.R.-R.); estibaliz.hernaez@ehu.eus (E.H.-L.); joseluis.vilas@ehu.eus (J.L.V.-V.); 2 BCMaterials, Basque Center for Materials, Applications and Nanostructures, UPV/EHU Science Park, 48940 Leioa, Spain; 3 Macromolecular Chemistry Group (LABQUIMAC), Department of Organic and Inorganic Chemistry, Faculty of Science and Technology, University of the Basque Country, UPV/EHU, Barrio Sarriena s/n, 48940 Leioa, Spain

**Keywords:** poly-l-lactide (PLLA), click reaction, anti-inflammatory activity

## Abstract

In the last few decades, surgical implants have been widely used to restore the function of damaged bones or joints. However, it is essential to receive antibiotic or anti-inflammatory treatment to circumvent significant problems associated, such as the colonization of the implanted surface by bacteria or other microorganisms and strong host inflammatory responses. This article presents the effectiveness of the copper catalyzed alkyne-azide cycloaddition (CuAAC) (“click”) reaction by the linkage of a fluorophore to the poly(L-lactide) (PLLA) surface. The results were analysed by means of X-ray photoelectron spectroscopy (XPS), contact angle and fluorescence microscopy. Moreover, this current work describes the covalent immobilization of the anti-inflammatory drug indomethacin on a PLLA surface. The CuAAC click reaction was selected to anchor the drug to the polymeric films. The successful bioconjugation of the drug was confirmed by XPS and the change on the contact angle.

## 1. Introduction

Nowadays, ligament and tendon reconstructions or other surgical implants used in medicine have become attractive procedures to restore the functionality of affected joints [1]. Annually around 1 million hips are replaced worldwide and more than 250,000 knee replacements are provided. In addition, 30% of hospitalized patients have one or even more vascular catheters [2]. Along with the implantation of a surgical device different host responses appear. For example, contact could induce a negative response that enhances protein adhesion on the material surface, blood coagulation and complement activation. Another drawback is the initial acute inflammatory, in this case neutrophils and monocytes are adsorbed in the site of inflammation [3,4]. Moreover, if there is no restoration of the tissue, the initial inflammation may become to a choric one and may develop a fibrotic encapsulation, which implies a premature failure of the implant [5,6]. The inflammation process of the biomaterial is mediated by a complex reaction, which involves protein adsorption, leukocyte recruitment and activation, secretion of inflammatory mediators, and fibrous encapsulation of the implant [7]. In order to reduce macrophage adhesion at early stage and to block pro-inflammatory cytokine release chemical and physical modifications of biomaterials and immunomodulatory approaches have been developed [8,9].

Depending on the size and the surface properties of the material, the initial response of the living components of blood to the biomaterial may vary [10]. It is believed that the endothelialization of the blood-contacting device surface may overcome the problem [11]. Therefore, it is essential a surface that enhances cell–material interaction and promotes the endothelialization at a shorter time, however the modified surface may accelerate the aggregation of platelets before the formation of an endothelial monolayer, because of that a previous surface anticoagulant and anti-inflammatory modification are required [12]. The ideal non-thrombogenic blood-contacting surface in blood vessels could be formed by a monolayer of healthy endothelial cells. The surface topography of materials, such as the presence of grooves, pores, or fibres, can also influence the behaviour of the cultured cells, for example, cell adhesion, orientation, movement, growth, and activation [13]. In the case of hemocompatibility regulation, it is essential to present a good regulation of blood-material interface in blood-contacting medical devices, such as vascular graft and stents, heart valves and intravascular catheters [14].

Metals and metal alloys, such as titanium and its alloys, are widely used on implants due to their biological security, biocompatibility and good mechanical strength. Nevertheless, thus organic coatings are frequently employed in order to enhance corrosion resistance, which is one of the main disadvantages of metals. In addition, these coatings show better thromboresistance, antimicrobial action, dielectric strength, wear properties and lubricity over metals [15]. Among organic coatings, polymeric ones are the most used strategy due to the fact that they are inexpensive to apply and environmentally friendly. Furthermore, polymer coating can modify the negative effects caused by Ti6Al4V implants, such as the stress shielding effects [16,17]. Additionally, the stress that it is transferred by synthetic biodegradable polymers to the damaged tissue has shown to allow tissue healing comparing to metal implants [17]. On the other hand, polymer coatings provide large surface area-to-volume ratios [17]. Beside their suitable mechanical properties, polymeric biomaterials present different surface properties, such as surface chemistry, hydrophilicity, surface energy and charge density and their interactions with living tissue are necessary to ensure biocompatibility. All these properties can be adapted to the needs of each specific application. For example, poly(l-lactide) (PLLA) is one of the most promising biodegradable polymers and it is derived from natural feedstock such as corn starch rice, potatoes and other natural resources [18,19]. The mechanical properties of PLLA are similar to those of synthetic polymers like polypropylene; in addition, raw materials for PLLA preparation are abundant and, consequently, PLLA is low cost [20,21]. Moreover, PLLA can offer some benefits to implantable devices as a coating due to its bioabsorbability and biocompatibility [22,23] and its hydrolytic degradation does not leave toxic by-products in the body fluid [24,25]. An increment of surface hydrophilicity by a simple functionalization with hydrophilic moieties could overcome the hydrophobicity that PLLA surface presents, in this way PLLA can be suitable for biomedical devices [26,27].

The low hemocompatibility represents the main disadvantage of these biomaterials (i.e., polyethylene terephthalate, polylactic acid, polycaprolactone and their nanofibers). Thus, a thrombus formation could occur on the material surface (namely, surface-induced thrombus formation) and may cause failure of the implanted material, vascular occlusion, heart attack and stroke [28]. Hence, it would be convenient to adhere to surface an anti-inflammatory agent in order to enhance hemocompatibility, which provides a faster endothelialization. As a consequence of the immobilization, a thombogenic type surfaces may be avoided and so do the inflammation. Therefore, significant research efforts have focused on modifying material properties using various anti-inflammatory polymeric surface coatings to generate more biocompatible implants [29,30].

In order to have a bioactive surface a previous surface modification is required, for that two types of modification can be applied: (I) physical changed such as topography; (II) chemical modification of the surface to obtain specific properties, for instance, by drug immobilization [31]. Therefore, several methods are known to improve the reactivity of the surfaces such as UV irradiation, ozone-induced treatment, plasma treatment or chemical modification, e.g., click chemistry. This last strategy has been found very useful to graft covalently drugs to the material because it does not compromise the bioactivity with additional structural restrictions. Among click reactions, the copper (I)-catalyzed alkyne-azide cycloaddition reaction is a well-known methodology for the conjugation of complex biomolecules to the surface of many materials, because of the fact that it can be carried out at room temperature and using water solvent and physiological-like conditions. Beside its tolerance to a wide reactant, the 1,2,3-triazole formed in the click reaction shows a similarity with the peptidic bond of proteins, but with a higher resistance over the enzymatic hydrolysis. In consequence, the immobilized drug is maintained covalently attached to the surface without loss of activity.

As it has been shown in previous works, click reactions led successfully a covalent immobilization of different drugs to a polymeric surface. For example, Aizpurua et al. [32] were able to adhere covalently an antibiotic called vancomycin to the glass port using a copper-catalyzed click reaction and plasma treatment. On the other hand, Weissleder and co-workers were able to attach carboxylated dextran to the surface of magnetic iron oxide nanoparticles via click reactions. After the modification of carboxylated dextran, which contains either alkynes or azide functional groups, the derivatized dextran reacted with different biomarkers (biotin), fluorescence markers (indocyanine, fluorescein), steroid (oestrogen) and pharmaceuticals (paclitaxel) with the copper-catalyzed azide-alkyne (CuAAC) method [33].

The present study focuses on the covalent immobilization of indomethacin reaction by CuAAC for enhancing anti-inflammatory activity on PLLA films. To the best of our knowledge, this work represents the first bioconjugation of this drug to a PLLA polymeric surface. These films could be suitable as a coating to metallic implants, such as titanium alloys, in order to minimize the drawbacks of this type of prosthesis. Besides improving an anti-inflammatory response on the film, we anticipate avoiding thrombus formation owing to the immobilization of the drug. Indomethacin (1-(*p*-chlorobenzoyl)25-methoxy-2-methylindole-3-acetic acid) is a nonsteroidal anti-inflammatory drug (NSAID) that shows very effective antipyretic, analgesic, and anti-inflammatory activity. Its NSAID chemical classification is an indole-acetic acid derivative. The mechanism of action of indomethacin is similar to that of other NSAIDs, and both the therapeutic and adverse event profiles of indomethacin are caused by decreased production of prostaglandins. NSAIDs inhibit cyclooxygenase (COX), preventing the production of prostaglandins from arachidonic acid [34]. On the other hand, in order to test the effectiveness of the approach, the proposed methodology was carried out employing a fluorophore based on dansyl to bond covalently to a PLLA surface by CuAAC. This fluorophore has been widely used for derivatization and detection of proteins [35,36] and for the determination of mercury in water [37,38] due to react rapidly with primary and secondary amino groups of amino acids, but very slowly with alcohols.

## 2. Materials and Methods

### 2.1. Materials

Poly-l-lactide (PLLA) (Corbion, Amsterdam, The Netherlands) was used to prepare membranes. Chloroform (>98% Macron Fine Chemicals, Gliwice, Poland), *tert*-butanol (^t^BuOH), ethanol (EtOH), methanol (MeOH, 98%), acetonitrinile (99%, Panreac, Darmstadt, Germany), dichloromethane (CH_2_Cl_2_, 98%, Macron Fine Chemicals), toluene (99%, Sigma Aldrich, St. Louis, MO, USA), *N*,*N*-dimethylformamide (DMF, 99%, Macron Fine Chemicals), acetic acid (Sigma Aldrich), deuterated chloroform (99.8% Sigma Aldrich) and Milli Q water were used as a solvents. Sodium hydroxide (99%, Panreac), *N*-(3-dimethylaminopropyl)-*N*′-ethylcarbodiimide hydrochloride (EDC·HCl, 98%), *N*-hydroxysuccinimide (NHS, 98%), triethylamine (<99.5%), propargylamine (97.5%), 3-bromopropylamine hydrobromide (98%), sodium azide (NaN_3_, 99.5%), copper (II) sulphate pentahydrate (Cu_2_SO_4_·5H_2_O, 98%), sodium ascorbate (98%), sodium sulphate anhydrous (Na_2_SO_4_, 96%, Panreac), *N*,*N*-diisopropylethylamine (DIPEA, 99%), *N*,*N*′-dicyclohexylcarbodiimide (DCC, 99%), 1-hydroxybenzotriazole hydrate (HOBT, 97%), O-toluidine blue (TBO, dye content 80%), dansyl chloride (>99%), indomethacin (>99%), thionyl chloride (>99%) from Sigma Aldrich were used as reactants. All the synthesized products spectra are summarized in Supplementary Information (Schemes S1–S4, Figures S1–S8).

### 2.2. General Procedure for the Synthesis of 3-Azidopropan-1-amine

3-Bromopropan-1-amine hydrobromide (10 mmol) was dissolved in water/acetonitrile (1:1, 15 mL) and NaN_3_ (40 mmol) was added. The reaction mixture was heated at 80 °C for 24 h. After cooling to room temperature, the solution was basified by addition of 2 M NaOH solution, and the mixture was extracted with dichloromethane (3 × 10 mL). Organic layers were collected, dried over Na_2_SO_4_ and the solvent was evaporated to obtain the target amine [39]. 3-Bromopropan-1-amine (2.28 g, 10.4 mmol) was then treated with NaN_3_ (2.78 g, 42.8 mmol according to general procedure to afford 3-azidopropyl-1-amine as a yellowish oil (0.77 g, 77%). ^1^H-NMR (CDCl_3_): δ (ppm) 1.29 (q, *J* = 6.6 Hz, 2H, CH_2_), 1.99 (bs, 2H, NH_2_), 2.33 (t, *J* = 6.6 Hz, 2H, CH_2_), 2.92 (t, *J* = 6.6 Hz, 2H, CH_2_); ^13^C-NMR (CDCl_3_) 31.5 (CH_2_), 38.5 (CH_2_), 48.8 (CH_2_).

### 2.3. Synthesis of Dansyl Derivatives

Dansyl chloride (2.0 g, 7.4 mmol) was dissolved in dichloromethane (20 mL) and 3-azidopropan-1-amine (0.75 g, 7.4 mmol) and triethylamine (0.75 g, 7.4 mmol) was added. The reaction was refluxed for 48 h. After cooling to room temperature, the solution was extracted with dichoromethane (3 × 10 mL) and washed with a saturated solution of sodium chloride (2 × 10 mL). Organic layers were collected, dried over Na_2_SO_4_ and the solvent was evaporated under vacuum to afford *N*-(3-azidopropyl)-5-(dimethylamino)naphthalene-1-sulfonamide (**2a**) as a brownish oil (1.8 g, 94%). ^1^H-NMR (CDCl_3_) 2.86 (s, 6H, 3xCH_3_), 3.05 (t, *J* = 5.7 Hz, 2H, CH_2_, H1), 3.27 (t, *J* = 5.7 Hz, 2H, CH_2_, H2), 4.15 (s, 2H, NH_2_), 7.16 (d, *J* = 7.5 Hz, 1H, CH_arom_, H9), 7.52 (dd, *J* = 7.5 Hz, *J* =7.5 Hz, 2H, CH_arom_), 8.27 (dd, *J* = 8.5 Hz, *J* =7.5 Hz, 2H, CH_arom_), 8.53 (d, *J* = 8.5 Hz, 1H, CH_arom_, H5); 13C-RMN (CDCl_3_) 26.8 (CH_2_, C2), 39.4 (CH_2_, C1), 44.1 (CH_3_), 48.4 (CH_2_, C3), 115.3 (C_arom_-H, C10), 118.7 (C_arom_-C, C8), 123.2 (C_arom_-H, C12), 128.5 (C_arom_-H, C5), 129.5 (C_arom_-H), 129.9 (C_arom_-C, C13), 130.6 (C_arom_-H), 134.5 (C_arom_-S, C4), 152.0 (C_arom_-N, C9).

Dansyl chloride (1.0 g, 3.7 mmol) was dissolved in dichloromethane (20 mL) and propargylamine (0.237 mL, 3.7 mmol) and triethylamine (0.75 g, 7.4 mmol) were added. The reaction was refluxed for 48 h. After cooling to room temperature, the solution was extracted with dichoromethane (3 × 10 mL) and washed with a saturated solution of sodium chloride (2 × 10 mL). Organic layers were collected, dried over Na_2_SO_4_ and the solvent was evaporated under vacuum to afford 5-(dimethylamino)-*N*-(prop-2-yn-1-yl)naphthalene-1-sulfonamide (**2b**) as a yellowish oil (0.95 g, 89%). ^1^H-NMR (CDCl_3_) 1.91 (t, *J* = 2.53 Hz, 1H, CH) 2.86 (s, 6H, 2xCH_3_), 3.77 (d, *J* = 2.53 Hz, 2H, CH_2_), 5.26 (s, 1H, NH), 7.16 (dd, *J* = 7.5 Hz, *J* = 0.54 Hz, 1H, CH_arom_, H9), 7.52 (m, 2H, CH_arom_), 8.29 (m, 2H, CH_arom_), 8.52 (d, *J* = 8.5 Hz, 1H, CH_arom_, H5); ^13^C-RMN (CDCl_3_) 33.2 (CH_2_), 71.2 (CH), 84.3 (Ctriple bond), 44.1 (CH_3_), 115.3 (C_arom_-H), 118.7 (C_arom_-C), 123.2 (C_arom_-H, C12), 128.5 (C_arom_-H), 129.5 (C_arom_-H), 129.9 (C_arom_-C), 130.6 (C_arom_-H), 134.5 (C_arom_-S), 152.0 (C_arom_-N)

### 2.4. Derivatization of Indomethacin: Synthesis of ***3a***

Indomethacin (2.1 g, 5.6 mmol) was dissolved in dichloromethane (20 mL) and thionyl chloride (0.8 g, 6.2 mmol) was added. The reaction was refluxed during 2 h. After that, the excess of thionyl chloride was removed under vacuum and 3-azidopropan-1-amine (0.6 g, 6.2 mmol) dissolved in dichloromethane (5 mL) was added to the mixture along with additional dichloromethane (15 mL). Then, the reaction was stirred at room temperature for 72 h. After that time, the reaction was stopped by adding water and dichloromethane, the phases were separated, and the aqueous phase was extracted with dichloromethane (3 × 20 mL) and washed with a sodium chloride saturated solution (2 × 10 mL). Then, organic layers were collected, dried over Na_2_SO_4_ and, finally, the solvent was evaporated under vacuum. *N*-(3-azidopropyl)-2-(1-(4-chlorobenzoyl)-5-methoxy-2-methyl-1h-indol-3-yl)acetamide (**3a**) was obtained as a yellowish oil (1.5 g, 71%). ^1^H-NMR (CDCl_3_): δ (ppm) 1.69 (m, 2H, CH_2_), 2.38 (s, 3H, CH_3_), 3.25 (m, 4H, CH_2_), 3.64 (s, 2H, CH_2_), 3.82 (s, 3H, OCH_3_), 6.21 (s, 1H, NH), 6.69 (dd, *J* = Hz, *J* = Hz, 1H, CH_arom),_ 6.84 (d, *J* = Hz, 1H, CHa_rom_), 6.88 (d, *J* = Hz, 1H, CHa_rom_), 7.46 (m, 2H, CH_arom_), 7.61 (m, 2H, CH_arom_); ^13^C-NMR (CDCl_3_) 31.5 (CH_2_), 38.5 (CH_2_), 48.8 (CH_2_).

### 2.5. Preparation of PLLA Films

PLLA (30 g) was dissolved in chloroform (50 mL) for 2 h under magnetic stirring. The solution was finally precipitated in an excess of cold distilled methanol to isolate the product. Resulting materials were dried at 70 °C in vacuum for 48 h. Films were fabricated in a hydraulic hot press by compression moulding at 200 °C for 4 min under a pressure of 150 MPa followed by water quenching [40].

### 2.6. Hydrolysis of PLLA Films

20 mm × 10 mm rectangular PLLA films were washed in methanol/water (50/50 vol.) solution for 150 min. Samples were dried overnight before surface modification. Hydrolysis was performed according to Lao et al. [41] PLLA films were immersed in 0.25 M NaOH at 52 °C under constant magnetic stirring during 30 min. Samples were removed and washed in distilled water for 90 min and were further dried at 30 °C before characterization.

### 2.7. Amidation of PLLA Films

An acetic/acetate buffer solution of pH 5 was previously prepared and NHS (0.015 g, 0.13 mmol) and EDC·HCl (0.025 g, 0.13 mmol) were added to it. Then, PLLA film was submerged in the solution under constant stirring for 4 h at room temperature. After, they were immersed in another pH 5 acetic/acetate buffer solution and heated at 40 °C for 2 h. Then, another portion of EDC·HCl (0.028 g, 0.15 mmol) was added and films were left to react at room temperature for 24 h. After 24 h, they were washed in distilled water and dried at 30 °C under vacuum [40]. Once dried, the films followed two different pathways: in pathway A (PA) the films were immersed in a solution of 3-azidopropan-1-amine (2.00 g, 19.0 mmol) and in pathway B (PB) the films were modified with propargylamine (0.89 g, 16.1 mmol) for 24 h. Then, they were cleaned with water and finally they were dried under vacuum at 30 °C before characterization.

### 2.8. CuAAC Reaction on PLLA Films

A mix of ^t^BuOH/H_2_O 1:1 was previously prepared, either dansyl derivatives (**2a**, **2b**) or modified indomethacin (**3a**) were added to it along with CuSO_4_·5H_2_O (0.025 g, 0.10 mmol) and sodium ascorbate (0.025 g, 0.13 mmol). Once all the solution is homogeneous previously modified and dried amidated PLLA films are immersed on it during 72 h at room temperature. After 72 h, samples were washed in EtOH or MeOH with an ultrasonic bath in order to remove the excess of unreacted material. The reaction between dansyl derivative (**2a**) and propargylated PLLA was carried out through the pathway A (PA), whereas the reaction between dansyl derivative (**2b**) and PLLA with azide group was through pathway B (PB).

### 2.9. Characterization of the Samples

The amount of grafted carboxylic groups (–COOH) in the hydrolysis process was determined by a colorimetric method using TBO as a colorant. Chollet et al. [42] proposed this method which is based on an ionic interaction between the cationic colorant and the deprotonated carboxylate groups of the acid. Hydrolyzed PLLA films were immersed in a TBO 5 × 10^−4^ M basic solution for 5 min. Then, the films were removed and washed in a NaOH 2M solution, so that the excess of TBO could be removed. Subsequently the films were immersed in an acetic acid 50% aqueous solution in order to release the attached TBO and the solution was measured by UV-VIS (Shimadzu MultiSpec-1501 spectrophotometer, Kioto, Japan). The concentration of the carboxyl groups was determined using a calibration plot (Abs = 75,301 × M; R^2^ = 0.9984) by measuring the optical absorbance at 633 nm and based on the assumption that 1 mol of TBO reacts with 1 mol of carboxyl groups [43].

On the other hand, the change in hydrophobicity in PLLA films caused by surface modifications was analyzed using contact angle method (NEURTEK Instruments OCA 15 EC, Eibar, Spain). Milli-Q water was used as a testing liquid and sessile drop method (2 µL per drop) was carried out at room temperature to do the measurements. The average values were calculated using eight measurements of each composition.

The elemental analysis of modified PLLA films was carried out by X-ray photoelectron spectroscopy SPECS system (XPS, SPECS Surface Nano Analysis, Berlin, Germany) using focus monochromatic radiation source 500 with dual anode Al/Ag and it is equipped with a 150 1D-DLD analyzer (Phoibos, Berlin, Germany). PLLA samples were fixed with stainless steel holders and carbon tape during de measurements. Moreover, a carbon reference was used to do the measurements.

The fluorescence of the PLLA surface was analyzed before and after click reaction using an epifluorescence microscope Zeiss Axioskop (Jena, Germany).

The proton (^1^H-NMR) and carbon thirteen (^13^C-NMR) nuclear magnetic resonance spectra were performed at room temperature in an AV-300 spectrometer (300 MHz for ^1^H and 75.4 MHz for ^13^C) (Bruker, Rheinstetten, Germany) using deuterated chloroform as solvent. Chemical shifts (δ) are expressed in parts per million (ppm) relative to TMS using the residual signal of the solvent [7.26 ppm (1H) and 77.0 (13C)] as internal reference. Coupling constants (*J*) are expressed in Hertz (Hz).

## 3. Results and Discussion

### 3.1. Surface Hydrolysis and Amidation

**Scheme 1:**
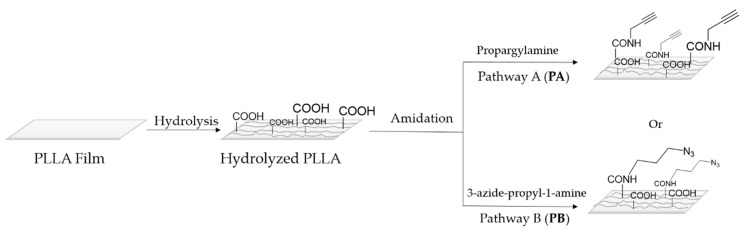
Representative scheme of hydrolysis and amidation reactions onto PLLA film through two different pathways.

**Figure 1:**
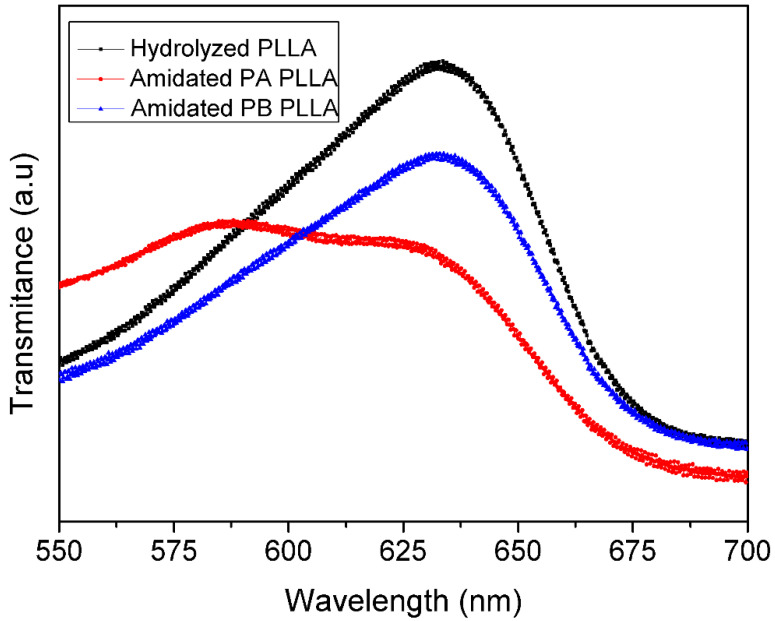
UV-Vis transmittance spectra of hydrolyzed PLLA and amidated PLLA.

The first step on the surface modification was a hydrolysis reaction and its subsequent amidation (Scheme 1). The amount of carboxylic group was quantitatively determined by a colorimetric method based on the ionic interaction between the TBO cation and carboxylate anions generated during PLLA basic hydrolysis (Figure 1). As can be seen in Scheme 1, after the hydrolysis, PLLA films were submitted to amidation conditions with propargylamine or, alternatively, with previously synthesized 3-azidepropan-1-amine. Therefore, it was proposed the conjugation of the fluorophore to the polymer surface through two alternative pathways. In pathway A, the alkyne functional group is linked to the surface. Whereas, in pathway B the azido moiety is the one linked to the polymer surface, and the alkyne to the fluorophore compound. After amidation conditions, colorimetric method was again employed, as amide group cannot be deprotonated, it cannot link with the cationic TBO, only unreacted acid group can interact with it indicating. Approximately 15% of the acid groups were successfully amidated with propargylamine by going through pathway A, while in pathway B the conversion of amidation was approximately of 7%. The amidation values were obtained by using the following calibration plot Abs= 75,301 × M; R^2^ = 0.9984, measuring the mol quantity of COOH group before and after amidation.

### 3.2. Synthesis Fluorophore Derivative

**Scheme 2:**
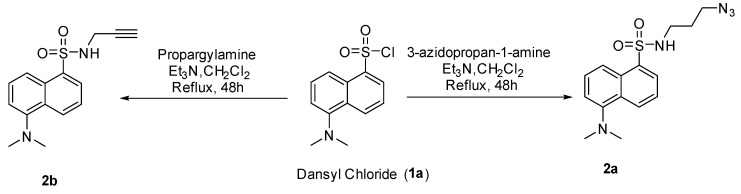
Synthesis of two different dansyl derivatives.

As commented, in order to find the most effective methodology, two alternative modifications were proposed on dansyl chloride, to carry out the two alternative click reactions the introduction of the alkyne or azide group into its structure was necessary (Scheme 2).

When the reaction was carried out at room temperature so as to obtain dansyl derivative **2a**, only the starting substrate was observed. Thus, the reaction time was prolonged to 48 h, however none promising result was obtained. Therefore, the temperature was increased until solvent reflux (40 °C). Fortunately, the heating allowed us to access to the corresponding sulfonamides **2a–b** with very good yields, 94% and 89% respectively.

**Scheme 3:**
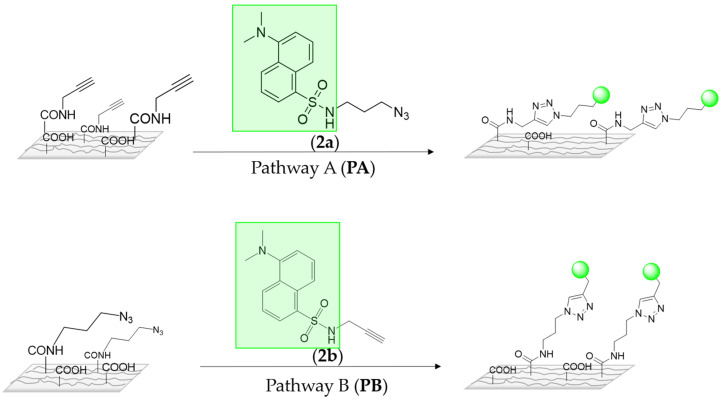
A schematic representation of two ways to immobilize the fluorophore derivative.

**Figure 2:**
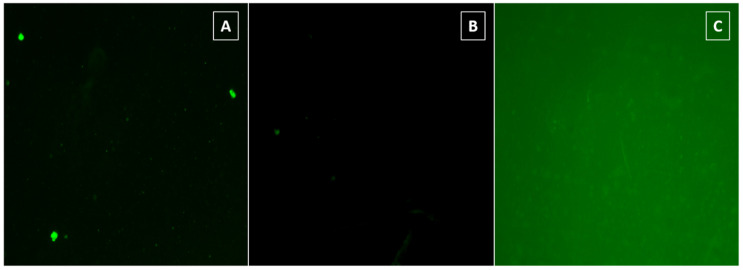
Representative fluorescence images of (**A**) hydrolyzed PLLA film, (**B**) conjugation of dansyl derivative **2b** onto PLLA film via PB, and (**C**) conjugation of dansyl derivative **2a** onto PLLA film via PA.

**Scheme 4:**
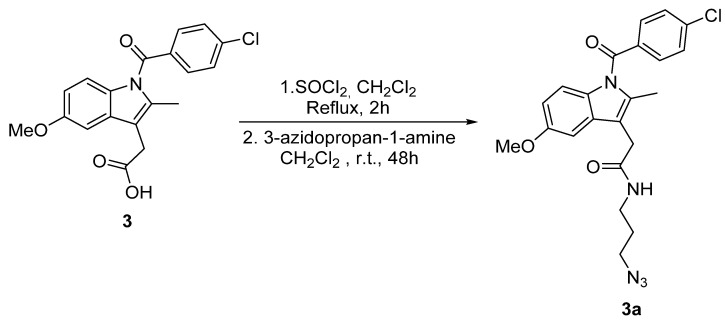
Synthesis of indomethacin derivative **3a**.

As it has been commented before, in order to monitor the azide alkyne click reaction, the surfaces were submitted to CuAAC clik reaction conditions with two different dansyl derivatives **2a–b** (Scheme 3), after that the surfaces were analyzed by means of a fluorescent microscope. As it can be seen in Figure 2, only the conjugation of dansyl derivative **2a** onto PLLA via pathway A showed an intense green fluorescence (Figure 2C), whereas fluorescent microscope revealed that the amount of dansyl derivative **2b** attached to the PLLA via pathway B was not enough to obtain a good fluorescence. Additionally, as it was expected, hydrolyzed PLLA did not show any type of fluorescence at the same power (20W). Consequently, these results revealed that the pathway A reaction, which implies the alkyne group linked to the polymeric surface and the azido to the modified fluorophore **2a**, is more effective to link drugs on PLLA. According to these results, indomethacin drug was proved to link to the surface via PA, with the azido moiety introduced on the drug (Scheme 4).

### 3.3. Derivatization of Indomethacin

In order to introduce an azido group into indomethacin structure several reaction conditions were tested by changing base, active agent, temperature and time (Table 1) (Scheme 4.)

As can be seen in Table 1, after several tests with different solvents, activating agent, base temperatures and times, only traces of the product were obtained in several reactions. (entries 16, 17, 20).

When dichloromethane was used as solvent, a standard amidation process was followed [44]. However, after several attempts in which the temperature, reaction time, activatinge agent and reagent equivalents were varied no product was detected. Therefore, it was concluded that it was necessary to increase the reaction temperature. As a consequence, it was decided to use other solvents with a higher boiling point, such as DMF or toluene. However, changing the solvent to DMF was unsuccessful, since again the starting substrate was obtained.

Finally, it was decided to test harsher reaction conditions. For that, a stronger activation agent—thionyl chloride—was used. The reaction was carried out in two steps; first, the chlorination of the carboxylic acid group which was carried out during 2 h in reflux, and then the amidation of 3-azidopropan-1-amine at room temperature for 72 h. Fortunately, the desire product was obtained with good yield.

### 3.4. Surface Chemistry and Morphology with Immobilized Indomethacin Derivative ***3a***

**Scheme 5:**
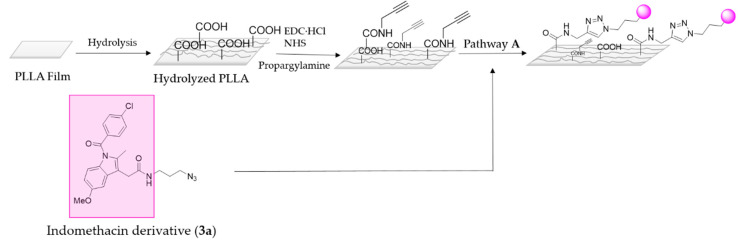
Representative scheme of indomethacin immobilization on PLLA film via pathway A.

Once indomethacin derivative **3a** was in hand its subsequent immobilization on the PLLA surface was performed following pathway A (Scheme 5).

**Figure 3:**
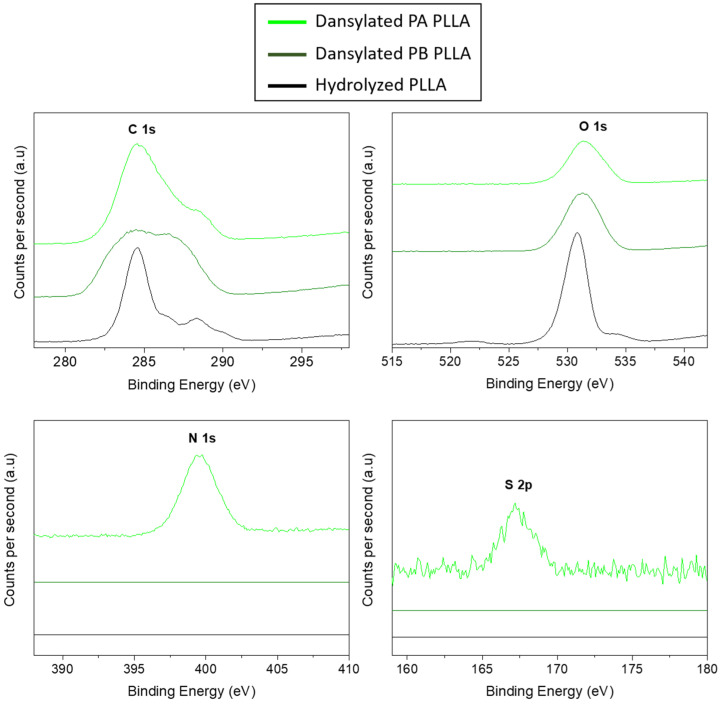
XPS spectra of different modified PLLA surfaces.

In order to evaluate the success of the proposed surface modification, the analysis of surface chemical composition of the PLLA surfaces were carried out by XPS before and after different reactions. The XPS spectra (Figure 3) corresponding to hydrolyzed PLLA films show two main contributions of C (1s) peak at 285 eV and O (1s) at 533 eV. After amidation and PA click reaction, an increase in the C (1s) peak can be clearly observed because of the attachment of more carbon-containing molecules. Moreover, small peaks corresponding to the contribution of nitrogen N (1s), with a binding energy of 395 eV, and sulphur S (2p), with a binding energy of 167 eV, are also observed in PLLA samples which with anchored dansyl derivative **2a**. However, in the PB click reaction with dansyl derivative **2b** onto PLLA neither nitrogen nor sulphur atoms appear. These results corroborated again, what was determined by the fluorescent microscopy, being the pathway A the most effective way to immobilize the fluorophore.

**Figure 4:**
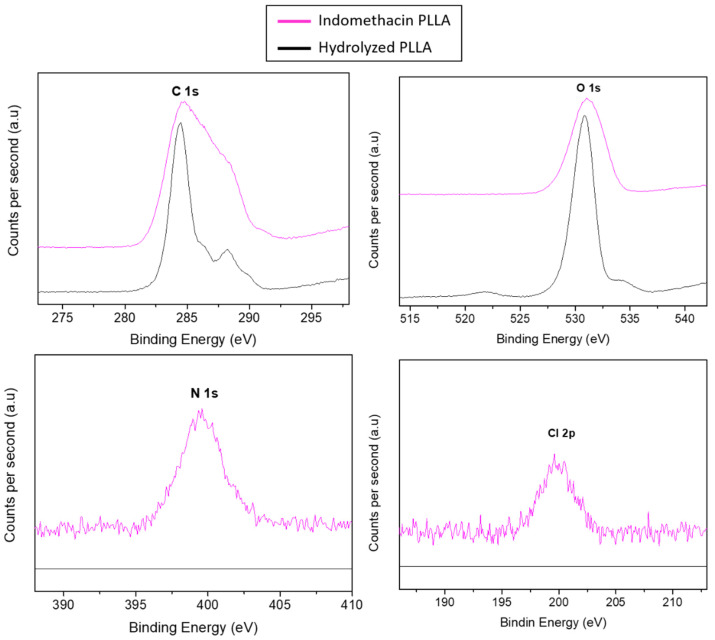
XPS spectra of indomethacin derivative **3a** immobilized on PLLA film.

Once the efficacy of the proposed methodology was tested, the same process was employed with the drug. The results of the immobilization of indomethacin derivative **3a** were observed by XPS. In Figure 4, a small peak can be observed with a binding energy of 200 eV corresponding to chloride element of the indomethacin derivative **3a**.

The surface composition of each sample is summarized in Table 2. According to the chemical structure of PLLA, the hydrolyzed films show that the surface is mostly made by carbon (38.7%) and oxygen (46.4%). This result suggests that the amount of oxide functional groups on the surface is highly significant. As shown in the table, before click reactions PLLA films had neither nitrogen, nor sulphur or chloride elements, as it can be expected. After the immobilization of dansyl derivatives, only in pathway A (PA) appeared nitrogen (1,2,3-triazole) and sulphur (SO) elements. Nevertheless, in reaction pathway B (PB), appeared carbon and oxygen in a higher amount comparing to nitrogen, which suggests again that immobilization of dansyl derivative **2b** is not the most appropriate for this surface. As it is previously commented, the indomethacin drug was modified to link to the surface via PA. The content of these elements shows a success of CuAACreaction when the azido group is attached to the drug. In all samples, a content of silicon is shown which is caused by the contamination of the glass.

**Figure 5:**
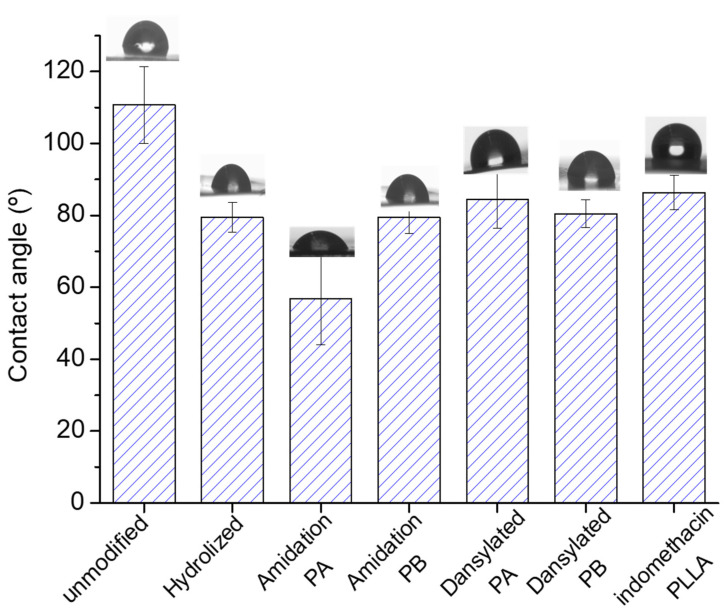
Water contact angles of untreated and modified PLLA.

Finally, through contact angle measurements the surface modification was also followed by analyzing any hydrophilicity changes induced in each step (Figure 5). As could be observed, the static water contact angle of untreated PLLA was 110°, which is highly hydrophobic and difficult for further surface treatment due to a lack of polar functional groups on the PLLA surface. The value of the contact angle decreased significantly to 78° after the hydrolysis process as a result of the presence of polar groups (–COOH). Subsequent to the amidation reaction with propargylamine, the contact angle decreased again. Nevertheless, the amidation with 3-azidopropan-1-amine did not drastically change the contact angle. Although the polarity of the attached functional groups diminished, the significantly important roughening of the surface could explain the decline in the contact angle value in case of the propargylation [45]. The covalent immobilization of both dansyl derivative **2a** and anti-inflammatory indomethacin enhanced contact angle. This result could be connected to the increase in hydrophobicity due to the presence of 1,2,3-triazole ring generated through the click reaction and the aromatics ring presented in both immobilized substances [46]. However, the covalent immobilization of dansyl derivative **2b** did not change too much compared to the propargylation process. These results suggested, again, successful modification of the polymer surfaces using pathway A, which is supported by the difference between the morphology of untreated and modified PLLA surfaces.

## 4. Conclusions

In this study, the effectiveness of the proposed methodology was proved by the attachment of a fluorophore and the consequent fluorescence observed in the modified films. Once the validation of the methodology was accomplished, indomethacin anti-inflammatory drug was covalently immobilized onto PLLA films via CuAAC click reactions. On the other hand, the successful bioconjugation of both substances, the fluorophore and the anti-inflammatory drug, was confirmed by the change on the contact angle and by XPS spectra. This PLLA surface permanently modified with indomethacin derivative could overcome or, at least, minimize significant problems associated with implants such as the formation of biofilm or blood clots and the inflammatory host response. It is noteworthy that the modification necessary to be anchored to the surface does not imply changes in the pharmacophore of the drug, so, the bioconjugation has been carried out without compromising the biological activity of the drug.

## Figures and Tables

**Table 1:** Reaction condition used for the amidation reaction of indomethacin.

Entry	3-Azido-1-propyl-amine (eq.)	Base (eq.)	Active Agent (eq.)	Solvent	T (°)	t (h)	Yield (%)
9	(1.5)	Et_3_N (1.5)	EDC·HCl (1.5)/HOBT (1.4)	CH_2_Cl_2_	Reflux	48	- [a]
10	(1.5)	Et_3_N (1.5)	EDC·HCl (1.5)/NHS (1.4)	CH_2_Cl_2_	Reflux	48	- [a]
11	2	-	DCC (2.1)	CH_2_Cl_2_	Reflux	48	- [a]
12	4	-	DCC (4.1)	CH_2_Cl_2_	Reflux	48	- [a]
13	5	-	DCC (5.1)	CH_2_Cl_2_	Reflux	48	- [a]
14	2	-	DCC (2.1)	Toluene	Reflux	48	- [b]
15	4	-	DCC (4.1)	Toluene	Reflux	48	- [b]
16	4	-	DCC (4.1)	Toluene	80	48	Traces [c]
17	1.5	DIPEA (1.5)	EDC·HCl (1.5)/NHS (1.4)	Toluene	80	48	Traces [c]
18	4	-	DCC (4.1)	DMF	85	48	- [b]
19	4	Et_3_N (4)	DCC (4.1)	DMF	85	48	- [b]
20	1	-	DCC (1)/DMAP (0.05)	CH_2_Cl_2_	r.t	24	Traces [c]
21	1	-	SOCl_2_(1.1)	CH_2_Cl_2_	Reflux/r.t	2+72	71

[a] Starting substrate was recovered; [b] decomposition was observed; [c] traces were observed by ^1^H-NMR along with starting substrate.

**Table 2:** The atomic weight percentage of hydrolysed and modified PLLA samples.

	Composition (%)					
Sample type	**C**	**O**	**N**	**S**	**Cl**	**Si**
Hyrolyzed PLLA	38.7	46.4	-	-	-	14.9
Dansylated **2a** PLLA (PA)	61.2	25.7	8.8	0.6	-	1.7
Dansylated **2b** PLLA (PB)	62.5	37.4	0.06	-	-	-
Indomethacin-PLLA	59.4	36.6	2.8	-	0.3	0.6

## Supplementary Materials

The following are available online at https://www.mdpi.com/2073-4360/13/1/34/s1, Scheme S1. Synthetic route of 3-azidopropan-1-amine. Figure S1. ^1^H-NMR spectrum of 3-azidopropan-1-amine. Figure S2. ^13^C-NMR spectrum of 3-azidopropan-1-amine. Scheme S2. Synthetic route of *N*-(3-azidopropyl)-5-(dimethylamino)naphthalene-1-sulfonamide (**2a**). Figure S3. ^1^H-NMR spectrum of *N*-(3-azidopropyl)-5-(dimethylamino)naphthalene-1-sulfonamide (**2a**). Figure S4. ^13^C-NMR spectrum of *N*-(3-azidopropyl)-5-(dimethylamino)naphthalene-1-sulfonamide (**2a**). Scheme S3. Synthetic route of 5-(dimethylamino)-*N*-(prop-2-yn-1-yl)naphthalene-1-sulfonamide (**2b**). Figure S5. ^1^H-NMR spectrum of 5-(dimethylamino)-*N*-(prop-2-yn-1-yl)naphthalene-1-sulfonamide (**2b**). Figure S6. ^13^C-NMR spectrum of 5-(dimethylamino)-*N*-(prop-2-yn-1-yl)naphthalene-1-sulfonamide (**2b**). Scheme S4. Synthetic route of *N*-(3-azidopropyl)-2-(1-(4-chlorobenzoyl)-5-methoxy-2-methyl-1H-indol-3-yl)acetamide. Figure S7.^1^H-NMR spectrum of *N*-(3-azidopropyl)-2-(1-(4-chlorobenzoyl)-5-methoxy-2-methyl-1H-indol-3-yl)acetamide (**3a**). Figure S8. ^13^C-NMR spectrum of N-(3-azidopropyl)-2-(1-(4-chlorobenzoyl)-5-methoxy-2-methyl-1H-indol-3-yl)acetamide (**3a**).
