# Supplementary material for: Poly(l-lactide)-Based Anti-Inflammatory Responsive Surfaces for Surgical Implants
Source: Polymers (Basel). 2020 Dec 24;13(1):34. doi: 10.3390/polym13010034 (PMC7794694; doi:10.3390/polym13010034)
Supplement: Supplementary file 1 [file polymers-13-00034-s001.pdf]

Supporting Information

PLLA Based anti-inflammatory responsive surfaces for surgical implants

Julia Sánchez-Bodón[1], Leire Ruiz-Rubio[1,2], Estíbaliz Hernaez-Laviña[1], José Luis Vilas-Vilela[1,2] and Mª Isabel Moreno-Benítez[3]

[1] Macromolecular Chemistry Group (LABQUIMAC), Department of Physical Chemistry, Faculty of Science and Technology, University of the Basque Country, UPV/EHU,Barrio Sarriena s/n 48940 Leioa, Spain; julia.sanchez@ehu.eus (J.S.-B.), leire.ruiz@ehu.eus (L.R.-R.), estibaliz.hernaez@ehu.eus (E.H.-L.), joseluis.vilas@ehu.eus (J.L.V.-V.)

[2] BCMaterials, Basque Center for Materials, Applications and Nanostructures, UPV/EHU Science Park, 48940 Leioa, Spain

[3] Macromolecular Chemistry Group (LABQUIMAC), Department of Organic Chemistry II, Faculty of Science and Technology, University of the Basque Country, UPV/EHU, Barrio Sarriena s/n 48940 Leioa, Spain; mariaisabel.moreno@ehu.eus (M.I.M.-B.)

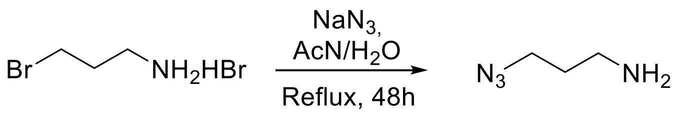

Scheme S1. Synthetic route of 3-azidopropanl-1-amine.

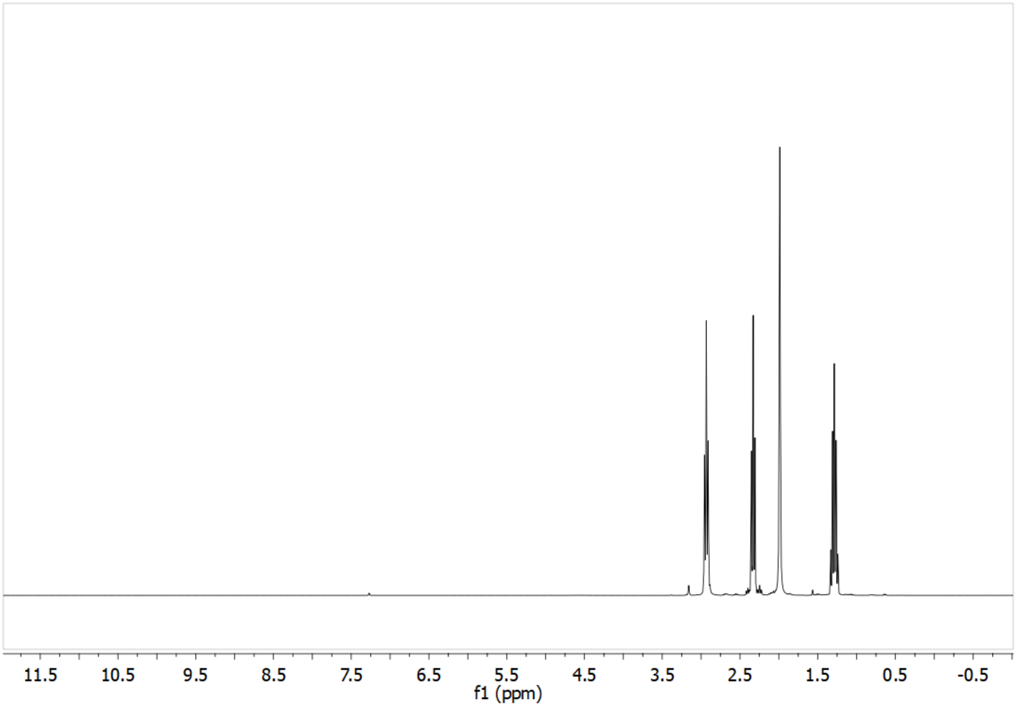

Figure S1. ^{1}H-NMR spectrum of 3-azidopropan-1-amine.

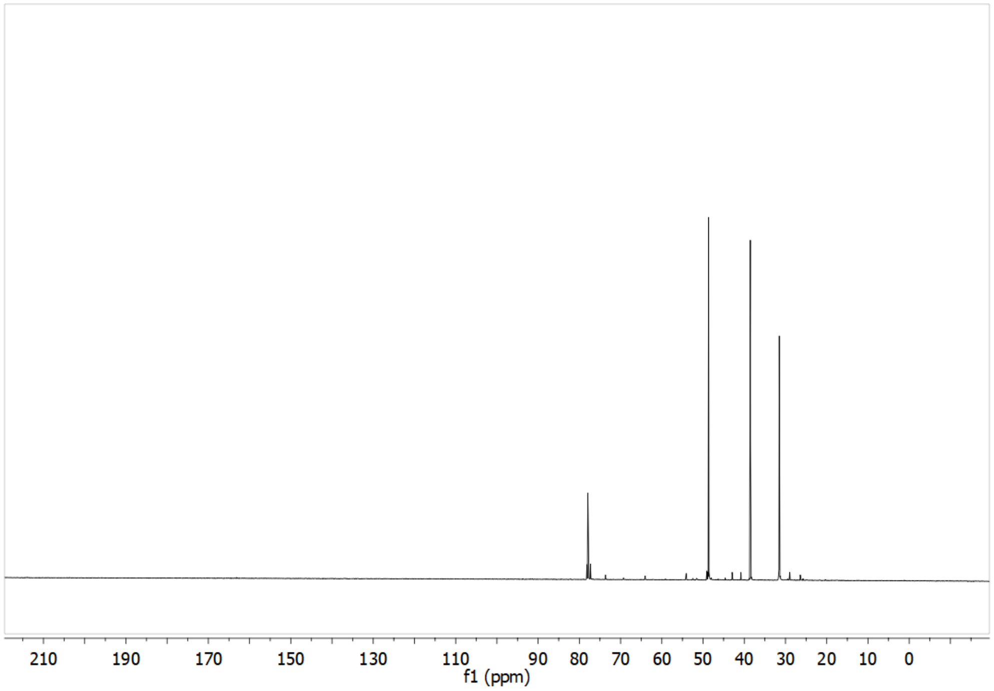

Figure S2. ^{13}C-NMR spectrum of 3-azidopropan-1-amine.

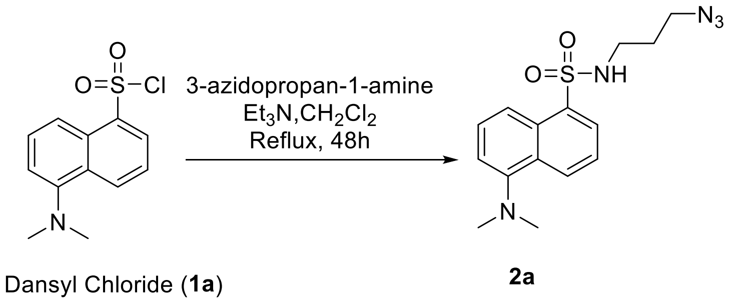

Scheme S2. Synthetic route of *N*-(3-azidopropyl)-5-(dimethylamino)naphthalene-1-sulfonamide (2a).

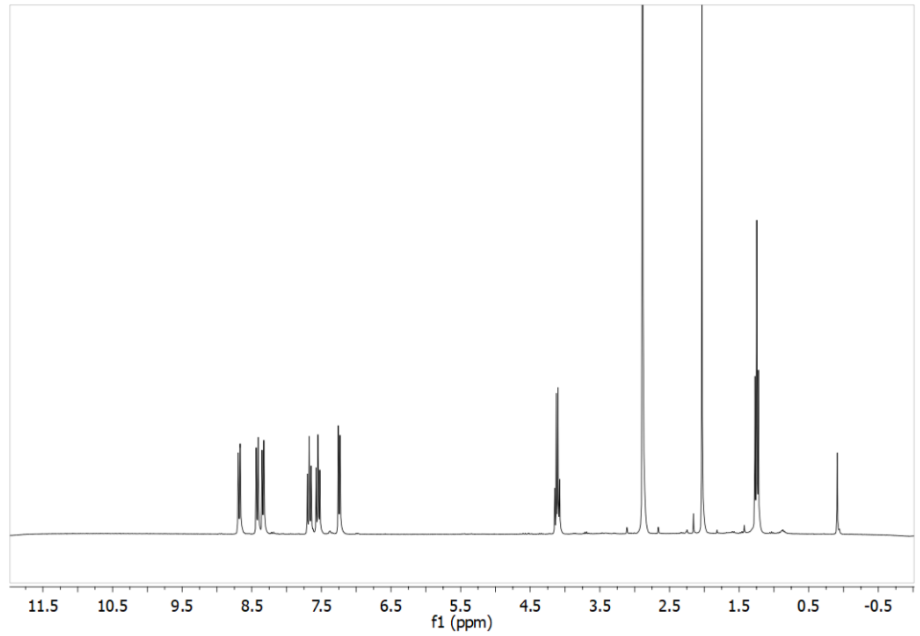

Figure S3. ^{1}H-NMR spectrum of *N*-(3-azidopropyl)-5-(dimethylamino)naphthalene-1-sulfonamide (2a).

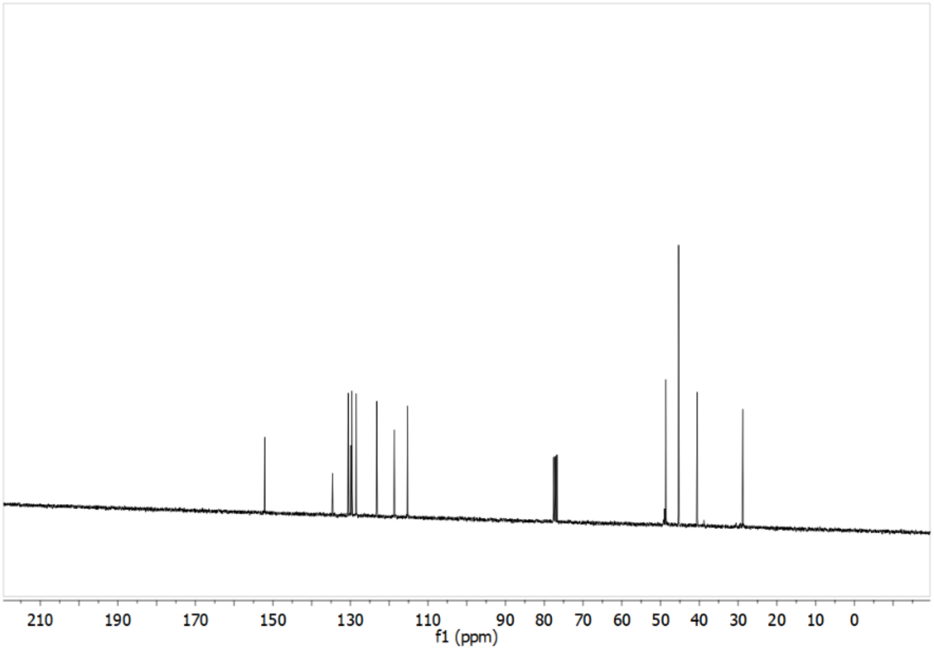

Figure S4. ^{13}C-NMR spectrum of *N*-(3-azidopropyl)-5-(dimethylamino)naphthalene-1-sulfonamide (2a).

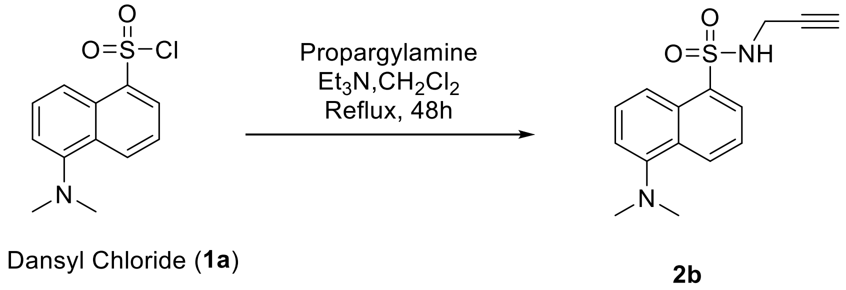

Scheme S3. Synthetic route of 5-(dimethylamino)-*N*-(prop-2-yn-1-yl)naphthalene-1-sulfonamide (2b).

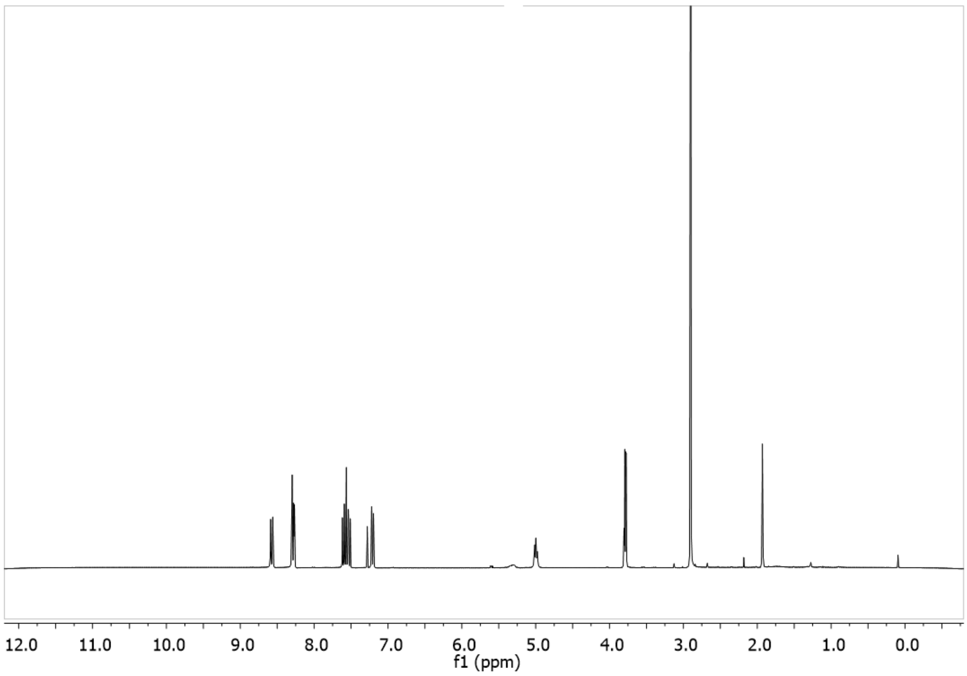

Figure S5. ^{1}H-NMR spectrum of 5-(dimethylamino)-*N*-(prop-2-yn-1-yl)naphthalene-1-sulfonamide (2b).

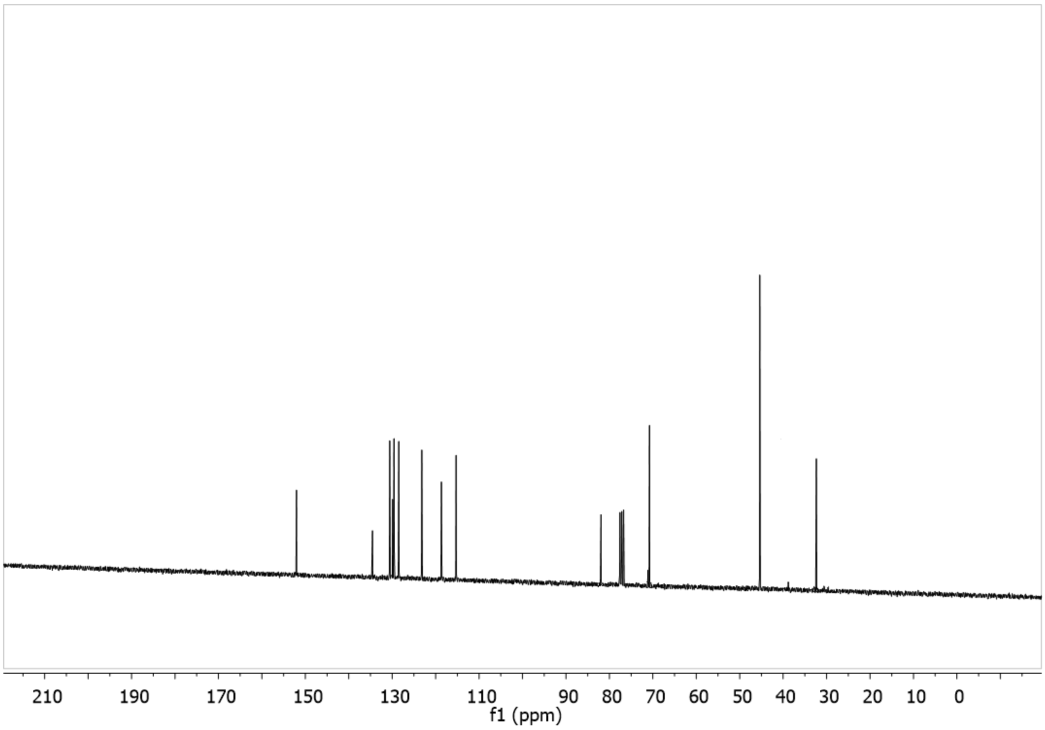

Figure S6. ^{13}C-NMR spectrum of 5-(dimethylamino)-*N*-(prop-2-yn-1-yl)naphthalene-1-sulfonamide (2b).

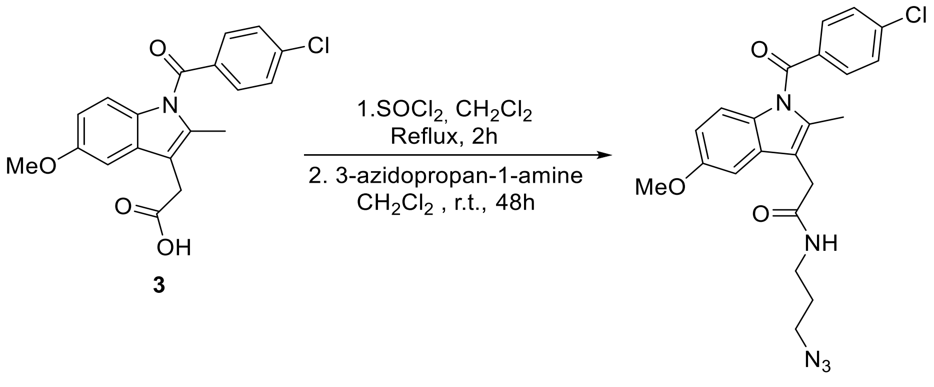

Scheme S4. Synthetic route of *N*-(3-azidopropyl)-2-(1-(4-chlorobenzoyl)-5-methoxy-2-methyl-1H-indol-3-yl)acetamide.

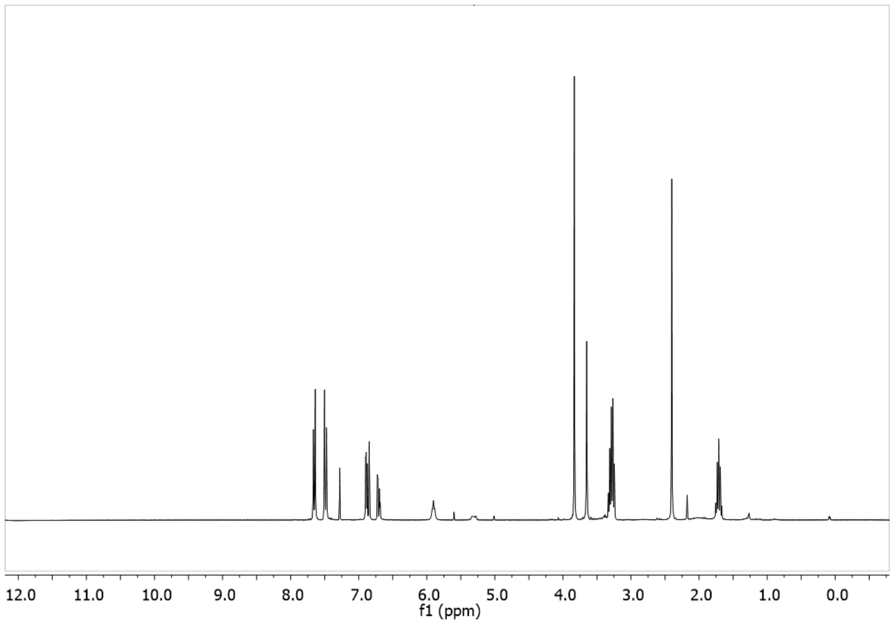

Figure S7.^{1}H-NMR spectrum of *N*-(3-azidopropyl)-2-(1-(4-chlorobenzoyl)-5-methoxy-2-methyl-1H-indol-3-yl)acetamide (3a).

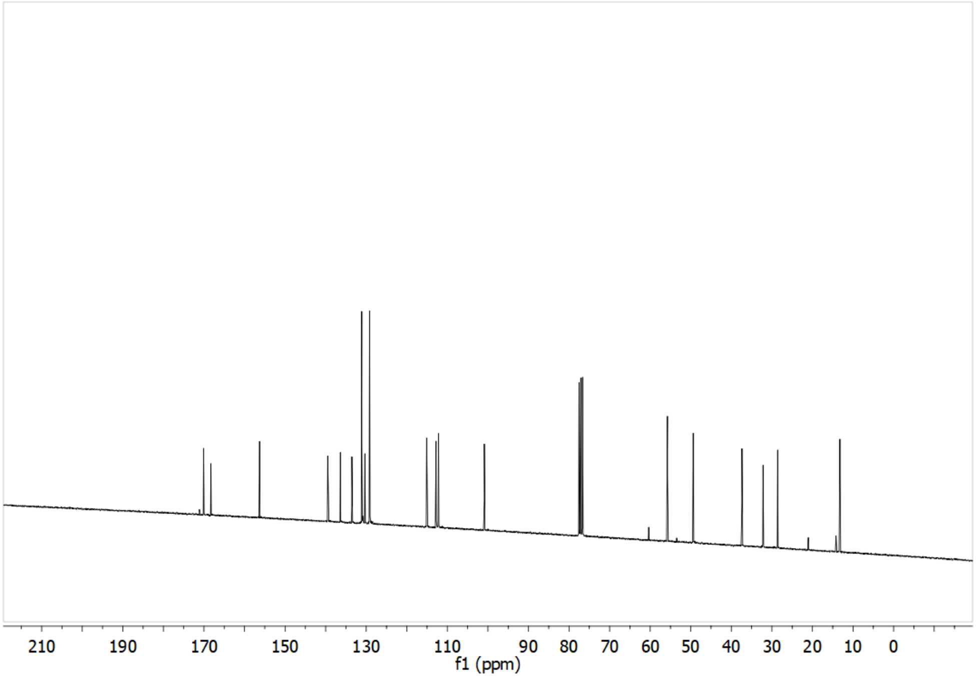

Figure S8. ^{13}C-NMR spectrum of *N*-(3-azidopropyl)-2-(1-(4-chlorobenzoyl)-5-methoxy-2-methyl-1H-indol-3-yl)acetamide(3a).
